# Medical Use of Over-the-Counter Canned Oxygen: A Review of Public Comments

**DOI:** 10.7759/cureus.79411

**Published:** 2025-02-21

**Authors:** Jestin N Carlson, Amelia J Carlson, Eunice Singletary

**Affiliations:** 1 Emergency Medicine, Saint Vincent Hospital, Erie, USA; 2 Education, Honors College, Millcreek Township School District, Erie, USA; 3 Emergency Medicine, University of Virginia, Charlottesville, USA

**Keywords:** first aid, over-the-counter, oxygen, oxygen support, oxygen therapy

## Abstract

Background: Over-the-counter (OTC) oxygen canisters are marketed to assist with recovery after exercise, alleviate mild altitude sickness, and be included in first aid kits, all without the need for a prescription. However, they are not approved for medical use. We describe the use of OTC non-medical oxygen for medical purposes, as reported through online reviews.

Methods: We reviewed a convenience sample of the most recent 200 online public reviews of the same non-medical canned oxygen product on two different popular web-based platforms (Amazon.com pulled on May 30, 2024, and TrustPilot.com pulled on June 2, 2024) for a single, commonly purchased OTC oxygen product. Two authors independently screened the reviews, extracted information, and assessed reported use for medical purposes (yes or no) and any response. Agreement on medical use was calculated using Cohen’s kappa. Medical reviews were categorized, and the frequency was reported.

Results: After excluding three reviews for lack of text, 48 of the 197 (24.3%) reviews were identified as being used for a medical purpose with 97% agreement between the reviewers. Medical reviews included terms such as shortness of breath/breathing (n=26), chronic obstructive pulmonary disease (n=11), and asthma (n=5). Reviewers reported the use of canned oxygen as a substitute for prescribed medical oxygen and breathing difficulty to avoid going to an emergency department.

Conclusion: Despite warning labels advising that the product is not approved for medical use, self-reported use included potential medical indications. Clinicians should advise patients of product limitations and potential risks when OTC oxygen is used as a substitute for medical oxygen and emergency care.

## Introduction

Over-the-counter (OTC) medical supplies improve patient access to therapies that may help alleviate suffering and treat minor ailments. One newer product includes OTC canned oxygen. These are concentrated oxygen delivery devices, available without a prescription, that deliver 95% oxygen in 1 s bursts through an attached facemask [1]. The product is supplied in 1-12 L cans and is advertised to help recovery after exercise and mild altitude sickness. These advertisements also suggest including OTC canned oxygen in first aid kits [1]. The canned oxygen market is expected to grow to over $9 billion annually by 2029 [2].

Non-medical, canned oxygen is not regulated by the U.S. Food and Drug Administration, and concerns have been raised regarding its use for medical conditions including reported cases of respiratory failure after usage [3]. As such, the potential benefits and harms (i.e., masking acute pulmonary conditions and delaying medical care) have not been rigorously studied and it is currently unknown if individuals are utilizing OTC canned oxygen for medical indications [4]. While oxygen may be necessary for certain respiratory conditions such as asthma or chronic obstructive pulmonary disease (COPD) exacerbations, steroids, and beta-agonists remain first-line therapies [5]. Delaying these therapies in exchange for OTC oxygen may place individuals at risk of worsening symptoms or respiratory failure [3]. Using publicly available reviews, we aimed to determine whether and for what potential medical purposes OTC oxygen is being used.

## Materials and methods

We employed a qualitative content analysis methodology to examine publicly available online customer reviews on Amazon.com and TrustPilot.com for the specific product category of “canned oxygen.” These sites were chosen as Amazon is the largest online retailer in the world [6], and TrustPilot is a free online review site with over 267 million online reviews [7]. Products identified in the initial search were further evaluated to identify a product sold over-the-counter as a single portable unit, without added scent, and with the greatest number of online consumer reviews. While many OTC oxygen products were available, we elected to examine the one meeting the above criteria and with a large portion of the market share (Milford, CT: Boost Oxygen) [8].

Eligibility criteria for including a review included readable textual review input, direct relevance to product use, and verified consumer purchase. Reviews by consumers who describe the use of the product on their children were included. We excluded any reviews that provided a score without textual input and without an English translation. No personally identifiable information was available for any reviews. The 100 most recent reviews were downloaded from Amazon.com via the Amazon Reviews Downloads extension for Chrome (Mountain View, CA: Google) on May 20, 2024, while the 100 most recent reviews from TrustPilot.com were downloaded using https://exportcomments.com on June 2, 2024. The https://exportcomments.com website allowed for the most recent 100 reviews. In order to ensure an equal number of reviews from sites, the 100 most recent reviews were collected from both sites.

Two reviewers (AJC and EMS) independently screened reviews, extracted information, and analyzed content. The analysis involved identifying recurring themes and patterns within the narrative text using thematic analysis, with identification of relevant medical history and product use through an iterative process to capture key consumer reasons for purchase and use of the product. Reviews describing use for a medical purpose were categorized by general themes, such as “history of asthma/COPD with shortness of breath.” Where available, we recorded consumer opinions about how they responded to the use of the product. We did not define "medical" use a priori as we wanted to broadly capture any review that could be considered medical. Therefore, an a priori definition of "medical" may have limited the number of cases considered medical. Each reviewer was allowed to use their own judgment to determine if the review described use for a medical indication and interrater agreement was calculated using Cohen’s kappa. Disagreements were discussed between the authors. As these data are publicly available, we did not seek institutional review board approval.

## Results

**Figure 1:**
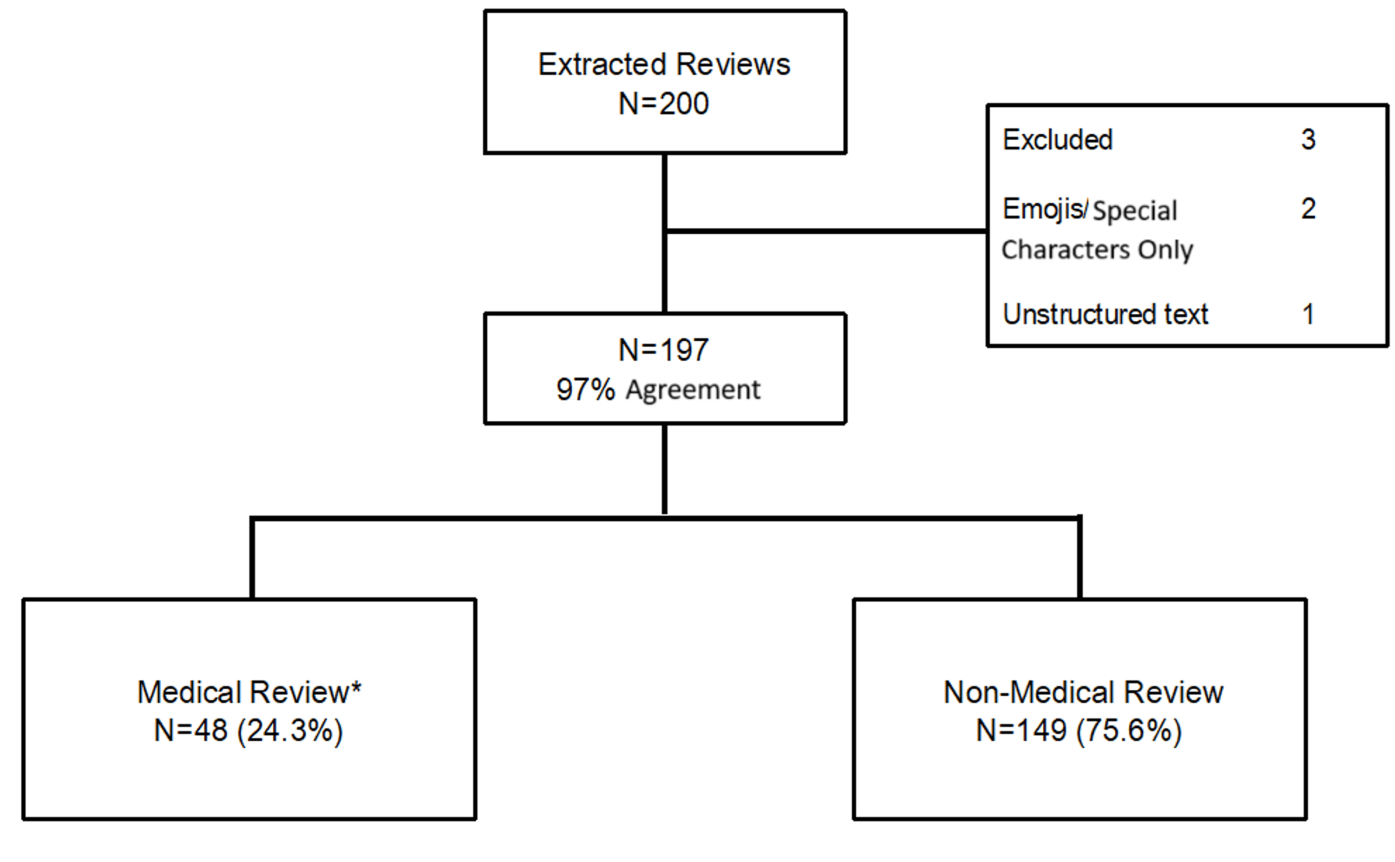
Diagram of included reviews and how they were classified in this study. *Considered medical by at least one reviewer.

The 100 most recent reviews from verified purchasers were downloaded on May 30, 2024. TrustPilot.com reviews were downloaded using https://exportcomments.com on June 2, 2024. At the time of data extraction, 2113 reviews were available on Amazon.com and 715 reviews were available on TrustPilot.com. Of the 200 extracted reviews, three reviews were excluded for lack of textual input (Figure 1). Of the remaining 197 reviews, 48 (24.3%) were identified by at least one reviewer as describing the use of the canned oxygen product for a medical purpose. Overall agreement between the two reviewers was 97% for medical vs. non-medical indications. Rating of the products was high across the reviews with 156 (79.2%) having either a 5 out of 5-star rating (n=129; 65.4%) or 4 out of 5-star rating (n=27; 13.7%).

The reviews considered the medical use of the product for the following: a specific medical condition (n=20), such as chronic obstructive pulmonary disease (COPD) and asthma exacerbation; as a substitute for home oxygen therapy or in cases of low oxygen levels (n=18); and for respiratory symptoms without a specified medical condition (n=18). Several reviews reported the use of OTC canned oxygen as a substitute for prescribed medical oxygen and to help avoid urgent medical care (Table 1). Specific comments include, “Works great if my oxygen blood level drops below 90%,” and “My son has chronic asthma. OTC canned oxygen has been a lifesaver for him and avoidance of having to go to the ER.” One reviewer reported using the product on his unconscious spouse and attributed her recovery before the arrival of emergency medical services to the use of the product.

**Table 1:** Reviews reporting over-the-counter (OTC) canned oxygen medical uses. *Total for all categories equals more than the total number of reviews deemed "medical" (n=48) as some reviews are applicable to more than one category. COPD: Chronic Obstructive Pulmonary Disease

Thematic grouping	Number of reviews*	Review examples
Used for care of symptoms related to a specific named disease or illness (e.g., COPD, asthma, pulmonary fibrosis, pulmonary hypertension, respiratory syncytial virus, obstructive sleep apnea, cluster headache, laryngospasm).	20	“I have pulmonary hypertension…normal (activity) leaves me breathless…(OTC canned) oxygen does the trick for me” “My husband has COPD, and he uses (OTC canned) oxygen 5-6 times a day” “…kept my fiancé out of the hospital when she had RSV…she (also) has asthma. “My son has chronic asthma. (OTC canned) oxygen has been a lifesaver for him and avoidance of having to go to the ER.” “I’ve been using (OTC canned oxygen) before I go to sleep. I have sleep apnea. “I have a vocal cord problem and sometimes my throat closes. I can use the oxygen boost to help till it stops or an ambulance comes.”
As a substitute for prescribed home or portable oxygen, or for low oxygen saturation readings.	18	“I am a lung cancer survivor…when I am not on portable oxygen…this gives me a bit of oxygen when my numbers are tanking” “I can… run into the store without carrying my portable oxygen unit” “for a quick trip out is better than packing my oxygen machine.” “Plenty O_2_ for short times away from the concentrator.” “I have COPD and it helps when I need a little extra oxygen.” “Works great if my oxygen blood level drops below 90%” “I have pulmonary fibrosis…if my (oxygen) levels drop too far I can use OTC canned oxygen.”
Used for care of respiratory symptoms (no underlying diagnosis provided): shortness of breath, difficulty breathing, breathlessness.	18	“…when you need that extra boost of oxygen when short of breath.” “…helps me with shortness of breath after going up 13 steps to second floor. I am 79 years old and overweight” “It really helps with my shortness of breath” “…provides quick relief from momentary shortness of breath. I have several of the large cans around my home” “It helps my husband when he can’t breathe.” “When I cannot breathe and my heart rate goes way up…” “I have serious breathing problems and with the [wild] fires my husband and I both use them all the time.”
Used for care of other symptoms: chest pain with loss of consciousness, headache, short-term memory loss.	3	“Without having the (OTC canned) oxygen on hand, I think she would have suffered brain damage or not survived.” “I travel with it and when the (cluster headache) attack occurs… I inhale 10 to 12 deep breaths of (OTC canned) oxygen. In a matter of minutes, the attack is aborted.” “This… oxygen gave me my short-term memory back.”

Other recurring themes included the use of canned oxygen for travel to high altitudes or for symptoms of altitude sickness (n=21), and for recovery following sporting events (n=7). One review even mentioned using canned oxygen while piloting non-pressurized aircraft - "I am a pilot. I fly at night and in the mountains of the southwest. Most smaller planes don’t have O_2_ systems, and it’s not something we use daily. Flying at night on a cross-country flight, a few hits off the boost are great, maybe even a lifesaver. At night, the effects are immediate. I can see clearly right away, even in bigger GA planes. This small, lightweight can is a great backup. On hot days when density altitude matters, even at seemingly low elevations, the air is thin. I've loved this for a couple of years now. I keep a three-pack in the hangar." Another reviewer noted using it for short-term memory loss: “This boost oxygen gave me my short-term memory back. I was forgetting things constantly, and then I started using this, and my memory came back within 5 minutes. It was such a relief.”

## Discussion

This study shows that individuals report utilizing non-medical, OTC canned oxygen for potential medical purposes. We used publicly available data from large retail and review websites to gain insight into how individuals may be using canned oxygen and address an important gap in the literature. While we noted that nearly 25% of reviews could be classified as a medical indication, the exact number may vary based on site, product, and population. Healthcare providers need to be aware that patients may be utilizing these products and counsel them regarding the potential risks and limitations. For example, many of the reviews that indicated use for medical purposes also mentioned preexisting conditions, such as asthma and COPD. While oxygen may be necessary in certain asthma or COPD exacerbations, steroids and beta-agonists remain first-line therapies [5]. Delaying these therapies in exchange for OTC oxygen may place individuals at risk of worsening symptoms or respiratory failure [3]. Some reviewers who noted their use of supplemental home oxygen commented that the canned oxygen was being used as a substitute for portable oxygen delivery devices when shopping or running errands. It is unclear how often individuals with these conditions utilize OTC canned oxygen and future work is needed to determine the prevalence of use among these individuals.

Although manufacturer websites suggest that the canned oxygen product should be available in "every first aid kit" [1], the data supporting this is limited [4], and OTC canned oxygen is not recommended for inclusion in first aid kits by the American Red Cross Scientific Advisory Council [9]. The impact of including such products in first aid kits is unclear and the benefits in improving health outcomes remain unknown. As an example, the comment, “My son has chronic asthma. OTC canned oxygen has been a lifesaver for him and avoidance of having to go to the ER.” suggests that individuals may be using OTC canned oxygen rather than seeking emergency care, which may put the patient at risk of respiratory compromise. Widespread use of OTC canned oxygen, without proper education and disclaimers, risks delaying necessary medical treatments for various conditions. Additional work is needed to understand the impact OTC oxygen has on individuals who utilize it for potential medical indications.

This study has several limitations. We utilized public reporting of use and the actual frequency of uses in medical scenarios is unknown. How the product was utilized and its impact on patient outcomes requires further study. We extracted the most current 100 reviews from two specific sites for a single product. Other products and review sites exist and while uses may vary across products, our work suggests individuals are using OTC canned oxygen for medical indications. Other products and review sites may note different indications for use. We did not include all potential canned oxygen products or similar products that may have been identified with related search terms (e.g., oxygen canister). We wanted to broadly capture any review that could be considered medical so did not define "medical" use a priori. While this could lead to possible bias, rigidly defining "medical" use could also lead to possible bias. As the public posting and reading these online reviews may not have a medical background and may interpret reviews as advocating for medical use, we wanted to keep the potential definition as broad as possible and therefore did not prescriptively define "medical" use a priori. We used two reviewers to broadly capture "medical" use although these may not be all uses. Future work may examine more sites with more reviewers or even prospectively follow known users to address their utilization patterns in real-time. It is possible that some reviews may overlap between the two websites, although we did not note duplicate textual inputs with data extraction. The reviews we identified were all in the English language likely indicating a select group of individuals. How OTC is used in other populations is unknown. Further research is needed to determine the potential benefits and harms of OTC canned oxygen use for medical indications (e.g., asthma).

## Conclusions

Despite warning labels on OTC canned oxygen cans advising that the product is not approved for medical use, self-reported use includes potential medical indications. Physicians should be aware of the availability of OTC canned oxygen products and advise patients with lung disease of the product's limitations, risks, and potential for harm when used as a substitute for medical oxygen and emergency care.
